# Occupational Hearing loss in Africa: An interdisciplinary view of the current status

**DOI:** 10.4102/sajcd.v67i2.700

**Published:** 2020-03-03

**Authors:** Katijah Khoza-Shangase, Nomfundo F Moroe, Anita Edwards

**Affiliations:** 1 Department of Audiology, Faculty of Humanities, University of the Witwatersrand, Johannesburg, South Africa; 2 South African Journal of Communication Disorders, Durban, South Africa

**Keywords:** Africa, current, context, hearing loss, interdisciplinary, occupational

Noise-induced hearing loss is 100% preventable if the collaborative stakeholders in the prevention process are fully committed to the process and implement effective measures timely. Audiologists have within their scope of practice the prevention of hearing loss and this needs to be at the forefront of all advocacy campaigns to prevent occupational hearing loss (OHL). In a systematic review by Moroe, Khoza-Shangase, Kanji and Ntlhakana (2018), where literature into the exposure to occupational noise in developing countries suggested that the prevalence of occupational noise-induced hearing loss (ONIHL) is still high, significant gaps in locally relevant and responsive evidence were identified. There is also evidence that the mining industry is aware of this epidemic; however, the efforts to curb ONIHL are currently unsuccessful. These authors explored and documented current evidence reflecting trends in the management of ONIHL in the mining industry in Africa from 1994 to 2016 through the use of the Preferred Reporting Items for Systematic Reviews and Meta-Analysis. Findings from this systematic review indicated that there is a dearth of research on the management of ONIHL in Africa. The limited research on the management of ONIHL focuses on some aspects of the hearing conservation programme pillars and not on all the pillars as suggested by some scholars in the field. Furthermore, they found that published studies had small sample sizes, thereby minimising their generalisation. This systematic review’s findings highlighted a need for more studies on the management of ONIHL in the mining sector, as evidence suggests that this condition in African countries is still on the rise; hence, there is the importance of this Special Issue, based on South Africa.

The South African Mine Health and Safety Council (MHSC, 2016) clearly articulates its goal as ‘every mine worker returning from work unharmed everyday: Striving for zero harm’. Moroe et al. (2018) argue that as far as ear and hearing health is concerned, this goal has not been realised despite the concerted efforts from the MHSC and the Chamber of Mines in South Africa. These authors present evidence that reveals the reality that approximately 73.2% of miners in South Africa are exposed to excessive noise surpassing the legislated occupational exposure limit of 85 decibel (dB), despite hearing conservation programmes (HCPs) implemented in the mining sector (Edwards, Dekker, Franz, Van Dyk, & Banyini, 2011; Strauss, Swanepoel, Becker, Eloff, & Hall, 2014). This is over and above exposure to other toxins including burden of diseases such as tuberculosis, which exacerbate the problem (Khoza-Shangase, 2019).

Evidence from numerous studies, which have been conducted to evaluate the effectiveness and efficacy of management of OHL within the South African mining industry, have yielded unfavourable findings, which indicate that significant efforts are still required to successfully manage OHL (Dekker, Edwards, Franz, & Banyini, 2011; Edwards & Kritzinger, 2012; Edwards, Milanzi, Khoza, Letsoalo, & Zungu, 2015). Well-integrated and comprehensive HCPs are those that acknowledge the multi-component nature of the interventions, comprising seven pillars: periodic noise exposure monitoring, engineering controls, administrative controls, personal hearing protection, audiometric evaluations, employee and management education, and training and record keeping (Hong, Kerr, Poling, & Dhar, 2013). Moroe (2018) argues for these interventions to be viewed as a form of complex interventions in order to be realistic about implementation.

Against this backdrop, the guest editors, Prof. Katijah Khoza-Shangase and Dr Nomfundo Moroe, who are both from Department of Speech Pathology and Audiology at the University of the Witwatersrand, embarked on this Special Issue entitled ‘Occupational hearing loss in Africa: An interdisciplinary view of the current status’ by inviting research papers from academics and researchers, specifically in the mining sector in Africa. This Special Issue presents current contextually relevant and responsive evidence that fills the gap in occupational audiology, while responding to the call for evidence to be Afrocentric. The goal of the Special Issue is to provide a comprehensive current status overview of OHL within the African context in order to contribute towards the industry’s ability to meet the elimination of hearing loss targets. This Special Issue therefore looks at challenges as well as possible solutions for the African context. Papers presented include original research and review papers. The papers address any of the following sub-themes in the field of OHL that are either directly or indirectly related to the main theme: (1) recent advances in the management of occupational noise, (2) barriers and/or facilitators to hearing conservation, (3) contextual factors influencing implementation of HCPs, (4) monitoring and evaluation factors in occupational noise, (5) other toxins contributing to OHL and (6) policy and legislation in the management of occupational noise.

Published evidence indicates that the prevalence of OHL is high in low- and middle-income (LAMI) countries, such as South Africa. This is a serious indictment as hearing loss is known to represent a heavier burden in LAMI countries than in high-income regions of the world because of the challenges faced by LAMI countries when compared with their developed counterparts. This is particularly concerning in a country of significant socio-economic inequity such as South Africa, where OHL can present a limitation on the kind of employment suitable for a person with a hearing impairment (Reid et al., 2006). Establishing the status of OHL and its management in LAMI countries is important if strategic planning about this occupational health challenge has to be systematic and successful. This challenge is applicable to other African countries covered in this Special Issue, such as Tanzania and Zimbabwe.

The article ‘Risk vs. benefit: Who assesses this in the context of occupational noise-induced hearing loss in the mining industry?’ starts off by highlighting important strategic indicators as well as important variables that the occupational health and audiology community needs to consider in order to plan more effective HCPs to prevent ONIHL. Although this paper is contextualised within the South African mining context, it is generalisable to most LAMI countries. The authors highlight the importance of regulatory authorities to hold the employers accountable for the elimination of ONIHL, and the important role that audiologists should play in conducting best risk–benefit evaluation of HCPs during the development and monitoring process.

The role of regulatory authorities is explored comprehensively in the article entitled ‘A critical analysis of current South African occupation health law and hearing loss’. The authors provide a comprehensive review of current South African occupational health law in relation to hearing loss. One of the important gaps in the law identified by the authors is the highly divided legal framework of occupational health and safety (OHS) in South Africa, which, according to the authors, perpetuates a monological ‘excessive noise-hearing loss’ paradigm that has serious implication for the rights of all workers to equal protections and benefits specifically with respect to occupational audiology healthcare. These authors assert that there is a need to harmonise OHS law and expand the scope of hearing protection legislation to include the full range of established and present ototoxic hazards to ensure that all workers receive equal protection and benefits from the law.

Findings presented in the paper ‘Chemicals, noise and occupational hearing health in South Africa: A mapping study’ support the aforementioned view. This paper answers the question, ‘what is the nature of knowledge about occupational ototoxic chemicals and/or noise exposure in South Africa?’, with findings clearly indicating that chemical exposures are only just beginning to be recognised as ototoxic in South Africa; therefore, regulations need to consider this as an important aspect of HCPs. The articles ‘Burden of disease: A scoping review of HIV/AIDS and TB in occupational noise-induced hearing loss’ and ‘Middle ear pathologies in adults within the mining industry: A systematic review’ argue that over and above chemical exposure as a compounding factor in OHL, considerations of burden of disease in otology and audiology should be viewed as critical as certain diseases can cause hearing impairment as a primary effect, as a secondary or opportunistic effect, or as a side effect of treatment options for that disease. When an employee is suffering from any such diseases, concomitant exposure to hazardous noise levels can present an even bigger challenge to successful HCPs, if the complex interactions are not taken into consideration in the conception, implementation and monitoring of HCPs. Furthermore, the article ‘Occupational noise and age: A longitudinal study of hearing sensitivity’ reminds the readers of the impact of age as an influencing factor, over and above the aforementioned factors, also in OHL. The focus on the employees is further seen in the article ‘Does occupational noise matter among manufacturing SME workers? Empirical evidence from Magaba, Mbare’, where the authors explore how occupational noise impacts attitudes towards occupational noise exposure, susceptibility to hearing loss and job performance. The article ‘Occupational noise-induced hearing loss in the mining sector in South Africa: Perspectives from occupational health practitioners on how mineworkers are trained’ places the employees in the hands of occupational health personnel (OHPs) by exploring these stakeholders’ perspectives regarding education and training of mineworkers on ONIHL and its impact on mineworkers’ health. Important contextual factors, such as linguistic and cultural diversity considerations, are suggested as critical if efficient training programmes for employees of HCPs are to succeed within the African context.

The role of the OHPs, with a special focus on the audiologist, is centralised in HCPs in the articles ‘Secondary data review of a South African platinum mine: Estimating miners’ risk for occupational noise-induced hearing loss’, ‘Classification of audiograms in the prevention of noise-induced hearing loss’ and ‘Feedback-based noise management matrix in action’. Accurate, comprehensive and efficient data capturing, management and use is key to any successful programme implementation and monitoring. Such data management relies on the use of accredited sensitive and valid measures as well as classification and criteria – with the data allowing matrix and models to be utilised in a complex intervention manner to plan, execute and monitor primary preventive programmes such as HCPs. For example, the article ‘Feedback-based noise management matrix in action’ demonstrates a basic static feedback model with its practical applications, such as estimating, monitoring and providing quantitative information to aid miners, mining administrators and policymakers in decision-making around HCPs.

The articles entitled ‘Recent advances in HCPs: A systematic review’ and ‘Engineering noise control for mines: Lessons from the world’ offer critical review and analysis of advances that require serious deliberation and consideration because they could be of benefit to LAMI contexts such as Africa, with a goal to learn from developed contexts in order to meet the noise elimination targets. The authors of these papers provide this evidence while interrogating the practicability and implementation possibilities within the South African or African mining industry. These advances are presented with careful consideration of the African context, allowing the readers to apply these in a contextually responsive manner. The article ‘South African hearing conservation programmes in the context of tele-audiology: A scoping review’ takes the importance of contextual relevance to conclusion in this Special Issue by offering strong recommendations about the application of tele-audiology as a service delivery model because of its particular relevance in resource-constrained settings, such as Africa. Because of the significant capacity:demand challenges in LAMI countries and the need for scaling up audiology professions’ management of HCPs as they fall under their scope of practice, the authors of this manuscript suggest careful consideration of tele-audiology as a platform to deliver services in these contexts. Tele-HCPs have been explored as one of the leading recommendations for increasing access to services within LAMI contexts.

The above papers have comprehensively addressed issues of policy and law, context, assessment, management, monitoring, as well as challenges and solutions in this first Special Issue on OHL within the African context in the history of the South African audiology profession. Overall, these papers highlight the following key issues: (1) the importance of translation of knowledge, laws and regulations into practice; (2) the value of collaborative work of all stakeholders for HCPs to be successfully implemented within complex interventions, with audiologists centrally placed; (3) the need to keep abreast with recent advances and their implementation where relevant and responsive contextually; (4) the importance of context analysis in programme implementation; and (5) the complex nature of OHL as well as the importance of managing this within a complex intervention framework.

The guest editors are deeply grateful to the National Institute for the Humanities and Social Sciences and the Consortium for Advanced Research Training in Africa for the financial support in producing this Special Edition.
